# Computational assessment of hemodynamics-based diagnostic tools using a database of virtual subjects: Application to three case studies

**DOI:** 10.1016/j.jbiomech.2016.11.001

**Published:** 2016-12-08

**Authors:** Marie Willemet, Samuel Vennin, Jordi Alastruey

**Affiliations:** a Division of Imaging Sciences and Biomedical Engineering, King׳s College London, St Thomas׳ Hospital, London, UK; b Department of Clinical Pharmacology, *King’s College London British Heart Foundation Centre,* St Thomas׳ Hospital, London, UK

**Keywords:** Arterial hemodynamics, 1D modelling, Pulse wave velocity, Central pressure, Aortic flow, Augmentation index

## Abstract

Many physiological indexes and algorithms based on pulse wave analysis have been suggested in order to better assess cardiovascular function. Because these tools are often computed from in-vivo hemodynamic measurements, their validation is time-consuming, challenging, and biased by measurement errors.

Recently, a new methodology has been suggested to assess theoretically these computed tools: a database of virtual subjects generated using numerical 1D-0D modeling of arterial hemodynamics. The generated set of simulations encloses a wide selection of healthy cases that could be encountered in a clinical study.

We applied this new methodology to three different case studies that demonstrate the potential of our new tool, and illustrated each of them with a clinically relevant example: (i) we assessed the accuracy of indexes estimating pulse wave velocity; (ii) we validated and refined an algorithm that computes central blood pressure; and (iii) we investigated theoretical mechanisms behind the augmentation index.

Our database of virtual subjects is a new tool to assist the clinician: it provides insight into the physical mechanisms underlying the correlations observed in clinical practice.

## 1 Introduction

Cardiovascular disease is a major cause of morbidity and mortality worldwide (Gersh et al., 2010). Early diagnosis in clinical routine allows the clinician to prevent development of the disease and to offer adequate treatment to the patient. This can be achieved through derivation of indexes describing the function of the heart and large vessels, such as the systolic/diastolic and pulse pressures, ankle-brachial index, and augmentation index (AIx) of central blood pressure. In recent years, emphasis has also been placed on the importance of arterial stiffness in the development of cardiovascular disease (Laurent et al., 2006). Indexes describing the latter include the pulse wave velocity (PWV), arterial distensibility, and stiffness index. Diagnosis can also be achieved by applying specific algorithms to clinical data. For example, the transfer function is a commonly used algorithm that allows the estimation of central pressure from a peripheral pressure measurement (Chen et al., 1997).

Deriving and validating these diagnostic tools on cohorts of patients is a challenging task. In addition to being expensive and time-consuming, in-vivo measurements are subjected to experimental errors, they are not always available at all sites of interest, and fluctuate with the condition of the patient. Most importantly, the estimated index can rarely be compared to its “true” value, since the latter is unknown.

In this study, we propose to use a new methodology based on numerical modeling to assess the accuracy of these diagnostic indexes and algorithms. Computational modeling has proven to be an efficient tool to represent arterial blood flow. In particular, one-dimensional (1D) models offer a good balance between accuracy and ease of computation. Several studies have shown their ability to predict arterial pressure and flow waveforms under healthy (Reymond et al., 2011, Mynard and Smolich, 2015) and pathological conditions (Steele et al., 2003, Willemet et al., 2013). Most of 1D modeling studies focus on patient-specific applications, trying to reproduce a single set of parameters and a unique hemodynamic state (Reymond et al., 2011, Huberts et al., 2013, Willemet et al., 2013, Alastruey et al., 2016). This work uses the “population-specific" approach presented in Willemet et al. (2015). Using 1D modeling and variations in cardiac and arterial parameters within healthy ranges, we generated a database of 3325 healthy virtual subjects presenting a diversity of hemodynamic, structural and geometric characteristics. For each virtual subject, all characteristics are known at every point of the systemic arterial tree, i.e. anatomical and structural properties, as well as pressure, flow and area waves at the larger arteries; therefore allowing the computation of the exact value of the diagnostic tool. The main assumptions of our 1D formulation are: (i) thin elastic incompressible arterial wall, (ii) 55-artery network, (iii) outflow from the left ventricle prescribed as inflow boundary condition at the aortic root, and (iv) RCR Windkessel models as outlet boundary conditions. We refer the reader to Willemet et al. (2015) for further details on the methodology followed to generate the database.

In this work, we used this new database approach to study the following three clinical problems: assessment of arterial stiffness through PWV (Section 2), validation of an algorithm for estimating central pressure (Section 3), and investigation of theoretical mechanisms behind AIx (Section 4). These examples illustrate the potential of our database of virtual subjects to assess computationally the efficiency of indexes and diagnostic tools based on pulse wave propagation, as highlighted at the end of each section. We then discuss limitations (Section 5), summarize our main results (Section 6) and provide some perspectives for future applications (Section 7).

## 2 Assess the accuracy of clinical indexes

### Case study: Estimation of pulse wave velocity

We investigated the accuracy of three different methodologies proposed to estimate PWV in clinical practice: (i) the foot-to-foot, (ii) loop, and (iii) sum-of-squares methods. We compared them with the theoretical PWV based on the Moens–Korteweg relation (Willemet et al., 2015).

#### 2.1 Foot-to-foot PWV

Foot-to-foot PWV (PWV_ff_) is the recommended method in clinical practice to estimate arterial stiffness (Laurent et al., 2006). By computing the time delay between the feet of pressure or flow signals at two arterial sites, PWV_ff_ provides an average stiffness along an arterial segment. While most clinical devices compute PWV_ff_ using pressure signals acquired with tonometry or mechanotransducers (Laurent et al., 2006), new methods compute PWV_ff_ using flow waveforms acquired during magnetic resonance imaging (Ibrahim et al., 2010, Wentland et al., 2014). We aimed to assess the accuracy of these measured PWV_ff_ compared to the theoretical value within the wide variation of cases offered by the database.

##### 2.1.1 Method

Foot-to-foot PWV is computed as ∆L/∆t, with ∆L the distance travelled by the pulse wave, calculated as the difference between lengths of wave propagation from the heart; and ∆t the transit time between the feet of waveforms, computed using the intersection algorithm described in Gaddum et al. (2013). In this study, we limited our analysis to the PWV_ff_ along the aorta, i.e. from the aortic root to the iliac bifurcation. The corresponding theoretical PWV was computed using Eq. (2) from Willemet et al. (2015).

##### 2.1.2 Result

**Fig. 1:**
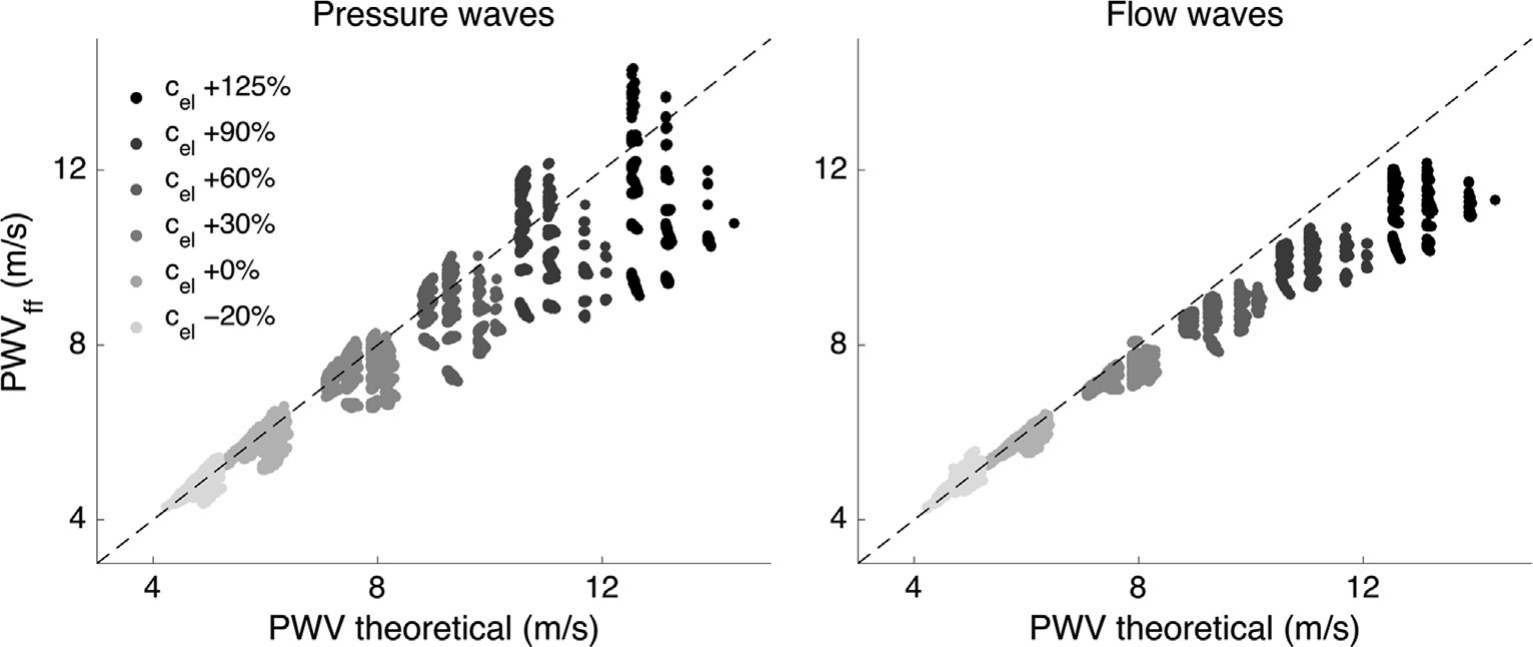
Comparison of theoretical and foot-to-foot PWV, PWV_ff_, along the aorta. Both foot-to-foot PWV (computed with pressure (left) or flow (right) waves) are reasonable estimates of the theoretical PWV along the aorta, i.e. from the aortic root to the iliac bifurcation. The larger the stiffness of the elastic arteries (c_el_), the larger the dispersion or deviation of the foot-to-foot PWV estimate. The dashed line indicates identity. The left plot presents the same results as in Fig. 6 from Willemet et al. (2015), and is reproduced here for ease of comparison.

Along the aorta, both pressure- and flow-based indexes reproduced the theoretical values relatively well, introducing a slight bias (underestimation) of −12% on average in stiffer arteries (Fig. 1). While flow-based PWV_ff_ tended to strictly under-estimate the theoretical PWV, pressure-based PWV_ff_ both under- and over-estimated the theoretical PWV, with a standard deviation twice larger than the flow-based one.

##### 2.1.3 Discussion

As detailed in our initial analysis (Willemet et al., 2015), this bias in PWV_ff_ is caused by reflected waves present during late diastole; these compromise the foot-to-foot algorithm assumption of a reflection-free period in early systole. These reflected expansion waves are due to negative reflection coefficients at the aorto-iliac bifurcation, which, according to Greenwald et al., 1990, are more likely to occur in older individuals.

Thanks to the virtual database, we could confirm that the aortic PWV can be estimated with good accuracy using the foot-to-foot method, either with pressure or flow signals.

#### 2.2 PU-, QA- and lnDU-loop PWV

The loop methods allow the estimation of the localized PWV from simultaneous measurements of two hemodynamic variables, by assuming that there are no reflected waves during early systole. Recent studies have shown that loop PWV indexes deviate from the theoretical PWV value quite significantly (Alastruey, 2011, Borlotti et al., 2014, Segers et al., 2014). We tested these indexes on all virtual subjects.

##### 2.2.1 Method

Loop PWV indexes were computed over the linear part of the loop during early systole (Khir et al., 2001, Rabben et al., 2004, Feng and Khir, 2010):

(1) $${\text{PWV}}_{\text{PU}}\text{=}\frac{1}{\rho }\frac{\text{d}P}{\text{d}U},\;{\text{PWV}}_{\text{QA}}\text{=}\frac{\text{d}Q}{\text{d}A},\;{\text{PWV}}_{\text{lnDU}}\text{=}\frac{1}{2}\frac{\text{d}U}{\text{d(ln}D\text{)}}$$

with ρ the blood density, *P* the blood pressure, *U* the blood velocity, *Q* the blood flow, *A* the luminal cross-sectional area and ln*D* the natural logarithm of the arterial diameter.

As suggested by Borlotti et al. (2014), in order to quantify deviations of the loop PWV from the theoretical PWV, we calculated the reflection coefficient at an arterial bifurcation:

(2) $$R_{t}=\frac{Y_{p}-Y_{d1}-Y_{d2}}{Y_{p}+Y_{d1}+Y_{d2}}$$

with Y=A/(ρPWV) the characteristic admittance of the parent vessel ($Y_{p}$) and daughter vessels ($Y_{d1}$*,*$Y_{d2}$).

##### 2.2.2 Result

**Fig. 2:**
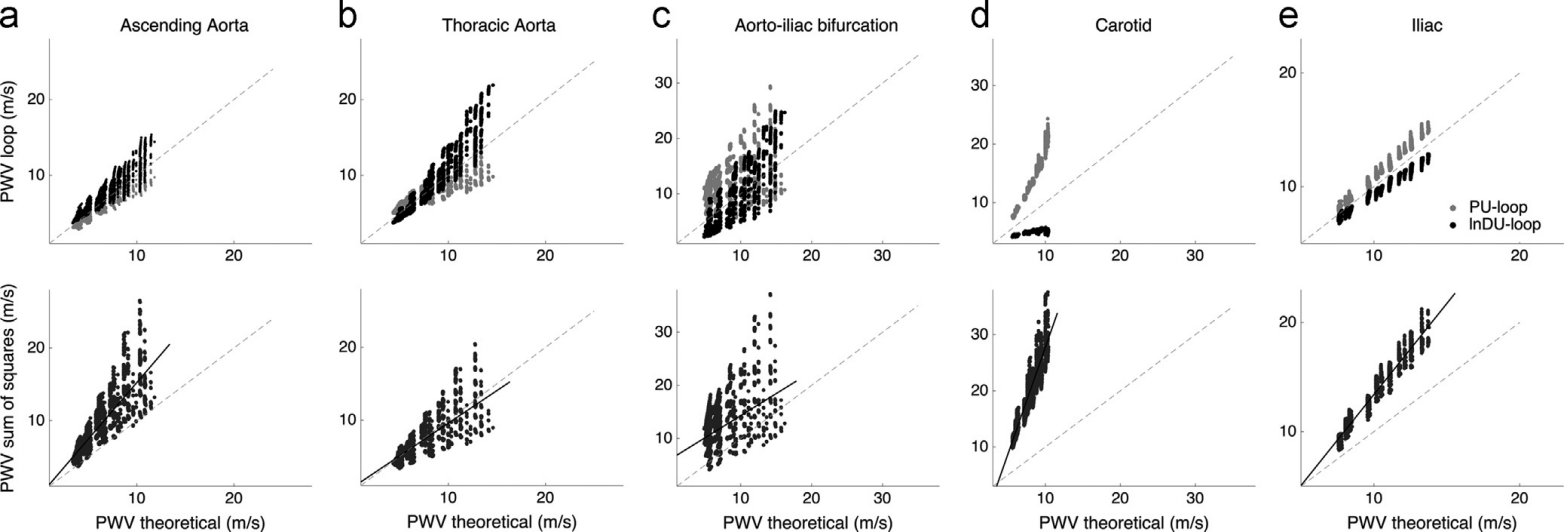
Comparison of theoretical PWV and local PWV estimates. The loop (top) and sum-of-squares (bottom) PWV indexes are presented at five arterial locations: (a) ascending aorta, (b) thoracic aorta, (c) aorto-iliac bifurcation, (d) carotid and (e) iliac arteries. The dashed line indicates identity. Top: PU-loop method in grey, lnDU-loop method in black. PWV estimated by the QA-loop superimposed on the lnDU-loop PWV and are not displayed here. Bottom: The continuous line is the linear fitting for each artery.

At the ascending aorta, the PU-loop PWV under-estimated the theoretical PWV by 7% while the lnDU-loop method over-estimated it by 10% on average (Fig. 2a, top). PWV loop estimates tended to invert along the aortic length towards the iliac bifurcation (Fig. 2b and c, top). In peripheral arteries, opposite deviations of the loop methods were observed: PWV_PU_ over-estimated the theoretical PWV, while PWV_lnDU_ under-estimated it (Fig. 2d and e, top). In the carotid artery, the PU and lnDU-loop errors increased up to +59% and −36% respectively, while in the iliac artery, these errors were about ±8%. In all arteries, PWV_QA_ was very close to PWV_lnDU_ with a slight over-estimation of less than 0.4 m/s.

We observed positive reflection coefficients *R*_*t*_ at the bifurcation downstream to the left common carotid artery, while negative *R*_*t*_ were observed for bifurcations along the aorta, leg and arm arteries. The aorto-iliac bifurcation presented reflection coefficients between −0.3 and +0.3.

##### 2.2.3 Discussion

In Alastruey (2011), the efficiency of local PWV methods was analyzed for a single virtual subject. Similar results were observed, though our results highlight the significant deviation of loop methods along the aorta for a wide variety of cases.

As suggested previously (Alastruey, 2011, Segers et al., 2014), using both lnDU (or equivalently QA) and PU-loop results can provide a more accurate PWV estimate. For example, taking the mean between lnDU and PU-loop PWV reduced the relative error to less than 4% for all arteries, at the exception of the carotid and aorto−iliac bifurcation where the error decreased to around +10%. Nevertheless, if pressure and area waveforms are available, one might simply compute the Bramwell-Hill PWV (Nichols and O’Rourke, 2005).

Borlotti et al. (2014) showed that loop methods were affected by positive and negative reflection sites: close to positive reflection sites, the PU-loop PWV over-estimated the exact wave speed while the lnDU-loop PWV under-estimated it, and vice-versa for negative reflection sites. In the arterial network, bifurcations (characterized by *R*_*t*_) are responsible for part of the reflections observed. The above theory is verified for the carotid artery, with positive *R*_*t*_: PWV_PU_ over-estimated the theoretical PWV while PWV_lnDU_ under-estimated it (Fig. 2d, top). Results along the aorta, with negative *R*_*t*_, were also in agreement with Borlotti׳s theory (Fig. 2a and b, top). The large variation of *R*_*t*_ at the aorto–iliac bifurcation might explain the inversion of the deviation of the loop methods towards the distal aorta (Fig. 2c, top). However, some peripheral arteries (e.g. iliac and brachial arteries) did not verify the above theory if arterial bifurcations alone are considered: *R*_*t*_ are negative, while the PU-loop PWV over-estimated (and the lnDU-loop PWV under-estimated) the theoretical wave speed (Fig. 2e, top). Additional sources of reflections, such as anatomical tapering and secondary re-reflections at bifurcations are not taken into account by *R*_*t*_ and might play an important role. The effect of positive reflections due to tapering needs to be verified in further studies.

Our results suggest that the loop methods should be used with care at arterial locations close to reflection sites.

#### 2.3 Sum-of-squares PWV (${\text{PWV}}_{\Sigma^{2}}$)

The sum-of-squares method was introduced to estimate locally the arterial stiffness by minimizing the net wave energy over a cardiac cycle, in short vessels where foot-to-foot methods cannot be applied (Davies et al., 2006). Initially introduced for the coronary arteries, its use in other arteries has been controversial since ${\text{PWV}}_{\Sigma^{2}}$ is computed over a period when waves are not unidirectional, and the resulting PWV is influenced by reflected waves (Alastruey, 2011, Kolyva et al., 2008).

##### 2.3.1 Method

The sum-of-squares method computes PWV using simultaneous pressure *P* and velocity *U* measurements as

(3) $${\text{PWV}}_{\Sigma^{2}}=\frac{1}{\rho }\sqrt{\frac{\sum \text{d}P^{2}}{\sum \text{d}U^{2}}}$$

with d*P* and d*U* the changes in pressure and velocity, respectively, across the wave fronts, and the sum extending during one cardiac cycle.

##### 2.3.2 Result

Fig. 2, bottom compares the sum-of-squares PWV to the theoretical PWV at five arterial locations: the ascending aorta, thoracic aorta, aorto-iliac bifurcation, carotid and iliac arteries. At the peripheral arteries, ${\text{PWV}}_{\Sigma^{2}}$ over-estimated the theoretical PWV by up to +140% (carotid) and +34% (iliac) on average (Fig. 2d and e, bottom). Along the aortic arch, there was also an over-estimation of the theoretical PWV by 50% (ascending aorta, Fig. 2a, bottom) and 20% (descending aorta, results not shown here) on average. The agreement at the thoracic aorta was better on average with an under-estimation of 2%, even though individual models deviated from the theoretical value by up to 60% (Fig. 2b, bottom). Similarly to the loop PWV indexes, on average, a large deviation (+58%) was observed at the aorto-iliac bifurcation (Fig. 2c, bottom). The over-estimation of ${\text{PWV}}_{\Sigma^{2}}$ was also observed at the other arteries of the database.

##### 2.3.3 Discussion

A detailed analysis based on the reflection coefficient (not displayed here) confirmed that the bias of ${\text{PWV}}_{\Sigma^{2}}$ was related to the amount of reflected waves in the arterial system. In view of these results, the sum-of-squares method should be used only with great care in clinical practice, as this method does not always estimate the PWV accurately and is strongly influenced by wave reflections.

**Table:** 

**Summary**
The virtual database enables us to:
– Test the accuracy of an index based on pulse wave propagation since its “true” theoretical value can be computed exactly. – Understand the possible bias of an index by testing it over a large variety of cases with known cardiovascular properties. – Compare central and peripheral indexes since simultaneous pressure and flow waveforms are available at different locations of the arterial tree.

## 3 Validate your algorithm without experimental error

### Case study: Non-invasive estimation of central blood pressure

**Fig. 3:**
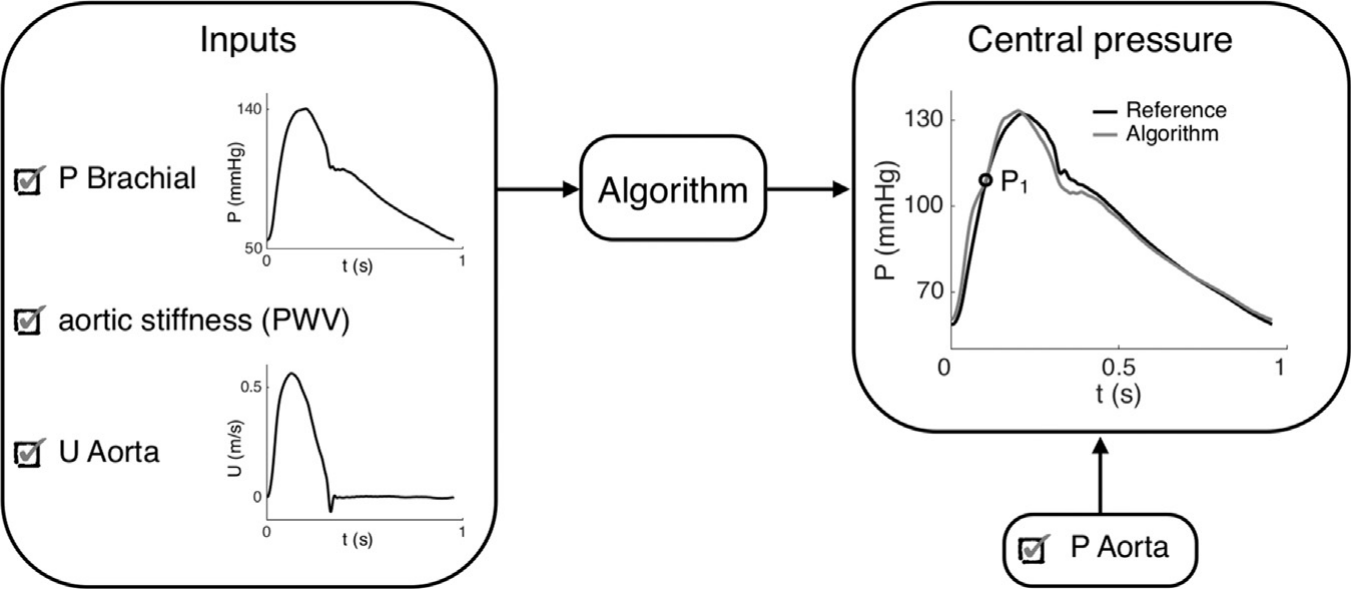
Schematic of the methodology of a recently proposed algorithm for estimating central blood pressure. Inputs include the brachial pressure waveform (*P*), the aortic velocity waveform (*U*) and the aortic stiffness, estimated by PWV. The output of the algorithm (the estimated aortic pressure waveform) is compared to the exact aortic pressure simulated using the 1D model for each virtual patient. The error between the systolic shoulders *P*_1_ of the estimated and reference central pressures is then computed.

Central blood pressure provides major information on the state of the heart and cardiovascular system. While there exists a number of non-invasive methods for estimating central systolic pressure (Chen et al., 1997, Guilcher et al., 2011), they are of limited accuracy and difficult to apply during cardiac magnetic resonance imaging. A new algorithm, which estimates the whole pressure waveform in the ascending aorta from non-invasive measurements, has recently been suggested (Vennin et al., 2015). This algorithm divides the target pressure waveform into an early systolic upstroke determined by the water hammer equation, a late systolic portion described by a second-order polynomial constrained by conditions of continuity and conservation of mean arterial pressure, and a diastolic decay equal to that measured in peripheral arteries. The algorithm uses the local aortic flow and stiffness, the mean and diastolic blood pressure, and the diastolic pressure decay to generate the central pressure waveform (Fig. 3). Thanks to waves being available at central and peripheral locations, our virtual database allows us to assess the efficiency of the new algorithm at reconstructing the aortic pressure waveform.

#### 3.1 Method

In the initial proof-of-concept study (Vennin et al., 2015), the aortic stiffness was evaluated using the sum-of-squares PWV at the ascending aorta (Eq. (3)). Because ${\text{PWV}}_{\Sigma^{2}}$ requires simultaneous measurements acquired invasively, in this study we evaluated alternative non-invasive methods to derive aortic stiffness: pressure-based carotid-femoral PWV (cfPWV_ff_) and brachial-ankle PWV (baPWV_ff_) acquired with tonometry, flow-based ascending to descending aorta PWV (adPWV_ff_) and descending to thoracic aorta PWV (dtPWV_ff_), lnDU- and QA-loop PWV at the ascending aorta, with distension and flow waveforms acquired during MRI (Wentland et al., 2014). In the central pressure algorithm of Vennin et al., (2015), the PWV mainly affects the systolic increase and specifically the estimation of the systolic shoulder *P*_1_ (Fig. 3). We therefore assessed the accuracy of *P*_1_ obtained from each PWV index by computing the error:

(4) $$\epsilon =P_{1}^{\text{algo}}-P_{1}^{\text{ref}}$$

where *P*_1_^algo^ is the systolic shoulder pressure estimated by the algorithm and *P*_1_^ref^ is the exact reference systolic shoulder pressure.

#### 3.2 Result

**Fig. 4:**
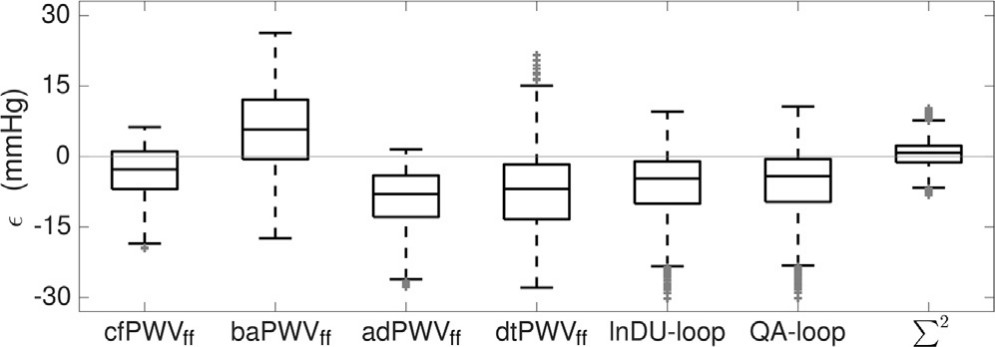
Distribution of the error in systolic pressure shoulder for different PWV methods. The error ϵ = *P*_*1*_^algo^−*P*_*1*_^ref^ between the reconstructed (*P*_*1*_^algo^) and the reference (*P*_*1*_^ref^) systolic pressure shoulders is computed using 7 different PWV methods. Each box indicates the 25th percentile, median, and 75th percentile; whiskers extend to minimum and maximum data points. Outliers are plotted in grey. cfPWV_ff_: pressure-based carotid-femoral foot-to-foot PWV; baPWV_ff_: pressure-based brachial-ankle foot-to-foot PWV; adPWV_ff_: flow-based ascending to descending aorta foot-to-foot PWV; dtPWV_ff_: flow-based descending to thoracic aorta foot-to-foot PWV; lnDU-loop, QA-loop and sum-of-squares ($\Sigma^{2}$) PWV were evaluated at the ascending aorta.

Results showed that the error on *P*_1_ was minimized if the sum-of-squares PWV was used (ϵ=0.6±2.8 mmHg, mean±SD) (Fig. 4). Within all non-invasive methods, the carotid-femoral PWV_ff_ provided the most satisfactory results with an average underestimation of *P*_1_ of -3.3±5.0 mmHg. Flow-based foot-to-foot PWV and loop methods under-estimated *P*_1_ with errors ranging from −5.5±6.7 to −8.8±5.9 mmHg. The baPWV_ff_, on the other hand, over-estimated *P*_1_ (5.7±7.8 mmHg).

#### 3.3 Discussion

In view of the deviation of the sum-of-squares method at the ascending aorta (over-estimation of 50% of the theoretical value, see Fig. 2a, bottom), this analysis highlights that the proposed algorithm introduces a bias in the estimation of the systolic shoulder, which is balanced when using the sum-of-squares method. Note that the database presents only type-A waveforms, with a systolic shoulder occurring before the systolic peak (Murgo et al., 1980). Furthermore, that shoulder is moderately marked. Therefore, the conclusions drawn here should only apply to that category of cases.

Given the wide collection of data available without experimental errors, the database has been a useful tool to help understand and improve quickly the methodology of a clinically relevant algorithm.

**Table:** 

**Summary**
The virtual database enables us to:
– Test an algorithm based on pulse wave propagation when accurate in-vivo data is not available. – Avoid inaccuracies from experimental error. – Refine an algorithm by testing different computational methodologies. – Obtain results quickly at no extra cost.

## 4 Understand theoretical mechanisms of wave propagation

### Case study: Investigating the augmentation index as a measure of wave reflections

The AIx is used clinically as a measure of wave reflection in the arterial network (Nichols and O’Rourke, 2005). It is assumed that the systolic shoulder visible on the pressure wave results from the arrival of the reflected wave interacting with the forward flow from the heart. Recently however, serious limitations about the validity of that concept have been suggested (Hughes et al., 2013), and it has been proposed that AIx may be indicative of the compliance of elastic arteries (Davies et al., 2010) and ventricular function (Fok et al., 2014). We assessed the relation between AIx and reflections using the database.

#### 4.1 Method

AIx was extracted from the pressure wave shape and was computed as the ratio of the augmented pressure (the pressure difference between the systolic peak and shoulder) to the pulse pressure. The shoulder point was detected by the zero-crossing of the fourth derivative of the signal, using the algorithm detailed in Segers et al., 2005, Segers et al., 2007, with a Savitzky–Golay differentiation filter of order 6 over 35 points. Reflections from the arterial network were assessed by decomposing the pressure wave into its forward (*P*_*f*_) and backward (*P*_*b*_) components, and by computing the ratio of backward to forward pressure peaks (*P*_*b*_*/P*_*f*_), as well as the timing of the backward peak. We also compared the AIx to the average reflection coefficient at all bifurcations of the network ($\bar{R_{t}}$), with each *R*_*t*_ computed using Eq. (2).

#### 4.2 Result

**Fig. 5:**
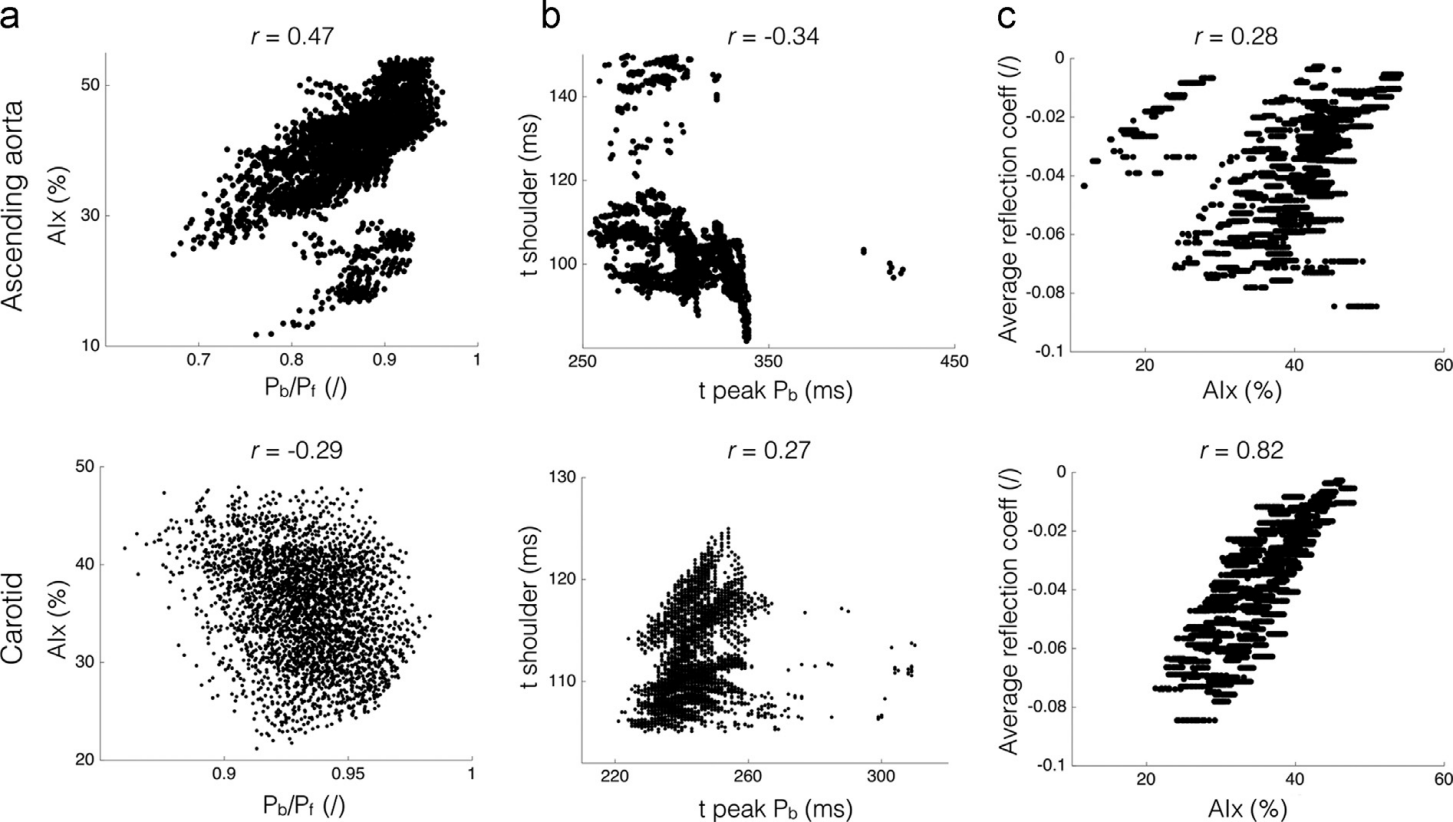
Relationship between augmentation index, AIx, and various indexes of arterial reflection at the ascending aorta (top) and carotid artery (bottom). (a) AIx vs ratio of backward to forward pressure peaks (*P*_*b*_*/P*_*f*_). (b) Timing of the systolic shoulder vs the timing of the peak of the backward wave. (c) Average of reflection coefficients at all bifurcations ($\bar{R_{t}}$) vs AIx. *r*: Pearson׳s correlation coefficient.

Results are presented at the ascending aorta and carotid artery (Fig. 5). At both arteries, AIx showed a weak to moderate linear correlation with the ratio of amplitudes *P*_*b*_*/P*_*f*_ (Pearson׳s correlation coefficient r=0.47 (aorta) and r=−0.29 (carotid), Fig. 5a). The timing of the shoulder point also only correlated weakly with the timing of the peak of the backward wave (r=−0.34 (aorta) and r=0.27 (carotid), Fig. 5b). However, at the carotid artery, we observed an important correlation (r=0.82) between AIx and $\bar{R_{t}}$, with AIx increasing as $\bar{R_{t}}$ tends to zero (Fig. 5c). This correlation was not as marked in the ascending aorta (r=0.28).

#### 4.3 Discussion

According to our results, AIx is not related to reflections when those are described through the decomposition into forward and backward pressure components. However, some correlation can be observed if one describes reflections by geometrical and structural properties of the arterial network. The difference between observed and theoretical reflections highlights the limited applicability to clinical data of the theory behind AIx. Indeed, as we are considering results from numerical modeling -without any experimental error-, one would expect much stronger correlations between AIx and reflection indexes, if AIx were a robust measure of wave reflection in the arterial network. As stated previously, our current database contains only type-A waveforms, with a moderate systolic shoulder occurring before the systolic peak. As highlighted by Hughes et al. (2013), different associations might be observed if type-C waveforms are studied.

While this analysis does not go into the details of providing a clear explanation of the origin of the augmentation index, it highlights that arterial wave reflections, as assessed in clinical literature, are not the only mechanism influencing the systolic shoulder. Interpreting the augmentation index as a measure of wave reflection only should therefore be made with caution.

**Table:** 

**Summary**
The virtual database enables us to:
– Understand physical mechanisms of pulse wave propagation. – Challenge established concepts, and highlight their limitations.

## 5 Limitations

The results presented in this study depend on the characteristics of the 1D model of arterial hemodynamics used to generate the database.

The model produced pressure waveforms which are representative of a healthy adult subject older than 30 years of age, rather than a young adult. These are type-A waveforms with an early systolic shoulder (Murgo et al., 1980). In future works, we plan on using an improved description of the wall elasticity, together with a more detailed model of the heart (Mynard and Smolich, 2015) in order to address that limitation and generate type-C waveforms as well.

Our model contains the 55 larger systemic arteries; it includes neither the coronary circulation or hand arteries, nor a detailed cerebral arterial network. Extending the arterial network to include more peripheral vessels is worth considering for the following two reasons. (i) Non-invasive measurements can be acquired at peripheral sites: e.g. volume pulse using photoplethysmography at the finger tip or on forehead arteries. (ii) It may improve the accuracy of signals at our current terminal vessels; e.g. the carotid arteries at the entrance of the complex network of cerebral arteries. Another improvement to the current model relates to the length of the arterial network. Arterial lengths could be varied uniformly to consider the influence of subject height.

Despite these limitations, the database offers a wide range of hemodynamic waveforms under physiological conditions. We believe that it is representative of a subset of the “real population”; this still needs to be demonstrated in a comparison with a cohort of human-derived data in future works.

## 6 Summary

We have presented a new methodology to assess theoretically the efficiency of diagnostic tools (indexes and algorithms) based on pulse wave propagation. It consists of a database of virtual arterial waveforms generated by a wide range of physiological, structural and geometrical properties. We have applied this new approach to three different tools used in the clinic to study hypertension.

Thanks to the availability of the “true” theoretical value of arterial stiffness for each virtual subject, we have studied the accuracy of pulse wave velocity estimates. According to our results, PWV estimates are affected by wave reflections. Nevertheless, the central foot-to-foot PWV seems to be the most accurate -and easier to use- in clinical practice. By running a new algorithm for central pressure estimation on all virtual subjects without any experimental error, we identified some recurrent bias in the proposed algorithm due to PWV. Finally, by applying wave separation analysis on central waveforms, we challenged the established concept that the augmentation index is an estimate of arterial wave reflections.

These case studies have highlighted the potential of the database approach to assess theoretically the efficiency of indexes and diagnostic tools based on pulse wave propagation. Furthermore, the database has allowed us to identify important mechanisms of wave propagation relevant to pulse wave velocity and augmentation index.

## 7 Perspectives

We believe that the database of virtual subjects can become a new useful tool for the clinician, providing insights into mechanisms important for the design of large cohort studies, and advice on the most relevant in-vivo data acquisition protocol. Indeed, if an index or algorithm fails to be validated within the theoretical framework of the virtual database, it would then be even less likely to perform well in a clinical setting.

Our database is also a very useful tool for biomedical students as it offers hemodynamic waveforms at various locations of the arterial tree on which one can apply post-processing algorithms and theoretically test hemodynamic concepts.

This study has focused on three particular clinical case studies, but future works could assess other clinical indexes (e.g. stiffness or ankle-brachial indexes), and evaluate new diagnostic tools such as patient-specific transfer functions (Hametner et al., 2015). Future extensions of the database could include diseased conditions such as peripheral arterial disease or heart pathologies.

The complete database is available for download on the project website: www.haemod.uk/virtual-database.html, while the numerical code used to run simulations and generate virtual cases can be found at www.haemod.uk/nektar.html.

## Conflict of interest

None.
